# β-Catenin/Smad3 Interaction Regulates Transforming Growth Factor-β-Induced Epithelial to Mesenchymal Transition in the Lens

**DOI:** 10.3390/ijms20092078

**Published:** 2019-04-27

**Authors:** Aftab Taiyab, Julie Holms, Judith A. West-Mays

**Affiliations:** Department of Pathology and Molecular Medicine, McMaster Health Sciences Center, Hamilton, ON L8N 3Z5, Canada; taiyab@mcmaster.ca (A.T.); holmsjp@mcmaster.ca (J.H.)

**Keywords:** cataract, TGF-β, β-catenin, Smad3, lens, EMT

## Abstract

Cataracts are the leading cause of blindness worldwide. Although surgery is a successful method to restore vision loss due to cataracts, post-surgical complications can occur, such as secondary cataracts, also known as posterior capsular opacification (PCO). PCO arises when lens epithelial cells (LEC) are left behind in the capsular bag following surgery and are induced to undergo epithelial to mesenchymal transition (EMT). Following EMT, LEC morphology and phenotype are altered leading to a loss of transparency and vision. Transforming growth factor (TGF)-β-induced signaling through both canonical, TGF-β/Smad, and non-canonical, β-catenin/Wnt and Rho/ROCK/MRTF-A, pathways have been shown to be involved in lens EMT, and thus PCO. However, the interactions between these signaling pathways in the lens have not been thoroughly explored. In the current study we use rat LEC explants as an ex vivo model, to examine the interplay between three TGF-β-mediated pathways using α-smooth muscle actin (α-SMA) as a molecular marker for EMT. We show that Smad3 inhibition via SIS3 prevents nuclear translocation of β-catenin and MRTF-A, and α-SMA expression, suggesting a key role of Smad3 in regulation of MRTF-A and β-catenin nuclear transport in LECs. Further, we demonstrate that inhibition of β-catenin/CBP interaction by ICG-001 decreased the amount of phosphorylated Smad3 upon TGF-β stimulation in addition to significantly decreasing the expression levels of TGF-β receptors, TBRII and TBRI. Overall, our findings demonstrate interdependence between the canonical and non-canonical TGF-β-mediated signaling pathways controlling EMT in the lens.

## 1. Introduction

Cataract, a loss in transparency of the ocular lens, continues to be the leading cause of blindness worldwide, affecting nearly 20 million people, with the incidence rising due to a global ageing population [1,2,3]. Primary cataracts can be divided into two major groups; non-fibrotic cataracts comprising nuclear and cortical cataracts, and fibrotic cataracts encompassing anterior sub-capsular cataracts (ASC). Fibrotic cataract or fibrosis of the ocular lens is primarily caused by epithelial to mesenchymal transition (EMT) of lens epithelial cells (LECs) that ultimately results in lens opacification and loss of vision. The procedure involving surgical replacement of the cataractous lens with an artificial intra-ocular lens (IOL) is the most widely practiced treatment for cataract. However, a major postoperative complication of this procedure is the development of secondary cataract, also known as posterior capsular opacification (PCO), which is prevalent in 20–40% of patients undergoing cataract surgery [4]. Like ASC, PCO involves EMT of LECs, which have remained on the anterior lens capsule following cataract surgery. The LECs that have transformed during PCO show enhanced capabilities of migration, evasion of apoptosis, production and deposition of extracellular matrix (ECM) components, and capsular wrinkling [5,6]. Key features of transformed lens epithelial cells include downregulation of epithelial cadherin (E-cadherin), F-actin polymerization and upregulation of α-smooth muscle actin (α-SMA), all of which are known EMT markers [5,7,8,9].

Transforming growth factor β (TGF-β), a major cytokine, has been shown to induce EMT-driven fibrosis [5] in many systems such as lungs [10], kidney [11] and heart [12]. Importantly, both active and latent forms of TGF-β have been detected in the aqueous humor of the anterior chamber of the eye, and have been implicated in EMT-mediated lens fibrotic disorders including ASC and PCO [13,14] in response to surgical or accidental injury [15,16,17,18,19]. A number of experimental model systems, including those using rodents, have further demonstrated the role of TGF-β in EMT-driven ASC and PCO. For example, incubation of whole rat lenses, in vitro, with active TGF-β resulted in plaque formation/opacification in the lens and disruption of normal lens morphology leading to the transition of lens epithelial cells (LECs) into a myofibroblast phenotype indicative of ASC formation [20,21,22]. Further, incubation of ex vivo rat and mouse lens epithelial explants with TGF-β generated spindle-shaped myofibroblasts that exhibited decreased E-cadherin, increased expression of α-SMA and increased accumulation of ECM [23,24,25].

TGF-β receptors I and II (TβRI and TβRII), in the presence of TGF-β, form an active heterocomplex resulting in active TβRI [26], which activates receptor-Smads (R-Smads) namely Smad2 and Smad3 that heterodimerizes and translocates to the nucleus to promote transcription of genes including EMT-inducing genes [27]. Indeed, the association of TGF-β with TβR II/Smad is known to be critical in the disassembly of adherens junctions resulting in increased stabilization of the cytoplasmic pool of β-catenin [28]. In the context of lens EMT, loss of Smad3 attenuates injury-induced EMT of lens epithelial cells in a TGF-β dose-dependent manner [29]. Further, overexpressing active TGF-β1 using lens specific TGF-β1-overexpressing transgenic mouse results in EMT of lens epithelium that was partially prevented by suppressing Smad3 expression [30]. In addition to canonical signaling, TGF-β also regulates EMT through Smad-independent non-canonical signaling pathways that have been linked with fibrotic lens pathologies [23]. In particular, administration of TGF-β to the lens epithelium results in activation of Rho/ROCK and β-catenin/cyclic AMP-responsive-element-binding protein (CREB)–binding protein (CBP) signaling pathways, and inhibition of either result in prevention of TGF-β-induced EMT in the lens [31,32]. A number of recent reports have demonstrated that the interaction between canonical and non-canonical TGF-β signaling, namely β-catenin and Smad3, is critical for TGF-β-induced EMT [33,34,35]. In addition, this interaction is required for nuclear translocation of myocardin regulated transcription factor A (MRTF-A), a known master regulator of EMT-associated cytoskeletal genes such as α-SMA [33]. However, the role of Smad3 signaling in conjunction with β-catenin and MRTF-A signaling pathways has not been explored during TGF-β mediated transition of lens epithelial cells.

Despite the growing evidence for the importance of non-canonical TGF-β signaling during lens EMT, there are no reports of crosstalk between canonical (Smad3) and non-canonical (β-catenin) signaling pathways during TGF-β-induced EMT in the lens. The current study focuses on understanding the interaction of TGF-β-stimulated Smad3 (canonical) and β-catenin (non-canonical) signaling during EMT of LEC explants. In particular, we seek to determine whether TGF-β-induced Smad3 activation is β-catenin dependent and necessary for nuclear translocation of MRTF-A, and if Smad3 is critical for delocalization of β-catenin during TGF-β-induced EMT in the lens. 

## 2. Results 

### 2.1. Smad3 Signaling Inhibition Blocks TGF-B-Induced EMT in Lens Explants

**Figure 1:**
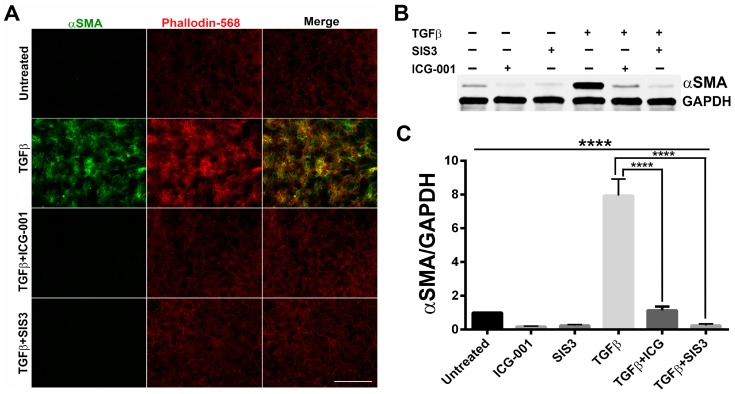
Inhibition of Smad3 blocks epithelial to mesenchymal transition (EMT). (**A**) Rat lens explants were treated with TGF-β in the presence or absence of SIS3 or ICG-001 (*n* = 3 where *n* ≥ 5 explants per treatment), used as a positive control, for 48h. Fixed explants were stained for α-SMA and F-actin/actin stress fibers and mounted with nuclear stain, DAPI. Images were acquired using 40X lens of Leica DM6 fluorescence microscope. Scale bars set to 100 µm. (**B**) Western blot was performed using protein isolated from rat lens explants (*n* = 3 where n ≥ 6 explants per treatment) incubated with TGF-β in the presence or absence of SIS3 or ICG-001 and was probed for α-SMA. GAPDH was used as a loading control. (**C**) Statistical analysis was performed using GraphPad Prism 6 that shows an 8-fold increase in α-SMA in TGF-β-treated lens explants, which was inhibited by SIS3 and ICG-001 (*p* < 0.0001).

As a downstream effector of TGF-β signaling, Smad3 is involved in the progression of EMT through its nuclear translocation in complex with other sister proteins such as Smad2 and Smad4, where they cooperate with DNA-binding transcription factors to activate or repress EMT-associated genes such as α-SMA [36]. To investigate the role of Smad3 in contributing to EMT in the lens, rat LECs (LECs) (*n* ≥ 5 per sample) were treated for 48 h with TGF-β in the presence or absence of Smad3 specific inhibitor, SIS3, targeting Smad3 phosphorylation [33,37]. Immunofluorescence analyses show an absence of α-SMA and actin stress (F-actin) fibers, the major markers of EMT in untreated LECs (Figure 1, panel 1). In contrast, TGF-β stimulated LECs showed an increase in both α-SMA and F-actin fibers (Figure 1, panel 2). However, the LECs co-treated with TGF-β and SIS3 showed attenuation of the TGF-β-induced increase in both α-SMA and F-actin, and a similar staining pattern to that of untreated explants indicating inhibition of EMT (Figure 1A, panel 3; *n* ≥ 5 per treatment). Further, our positive control, a β-catenin/CBP interaction inhibitor, ICG-001 [34], that has been reported to prevent TGF-β-induced EMT in LECs [32] also showed absence of α-SMA and F-actin staining in the presence of TGF-β (Figure 1A, panel 4; n ≥ 5 per treatment). In order to prove that our immunofluorescence staining for α-SMA translates to the actual protein expression, we performed western blots using proteins isolated from LECs treated with TGF-β in the presence or absence of SIS3 and ICG-001. Our western blot analyses revealed an ~8-fold increase in α-SMA in the LECs treated with TGF-β when compared to untreated LECs (*p* < 0.0001; *n* = 3 where *n* ≥ 6 per treatment). However, co-treatment of LECs with TGF-β and SIS3 resulted in a ~32-fold decrease in α-SMA expression when compared to TGF-β treated LECs (*p* < 0.0001; *n* = 3 where *n* ≥ 6 per treatment). In addition, LECs co-incubated with TGF-β and positive control, ICG-001, resulted in a ~23-fold decrease in α-SMA expression when compared to TGF-β treated LECs (*p* < 0.0001; *n* = 3 where *n* ≥ 6 per treatment). 

### 2.2. Smad3 Signaling Inhibition Blocks B-Catenin and E-Cadherin Delocalization

**Figure 2:**
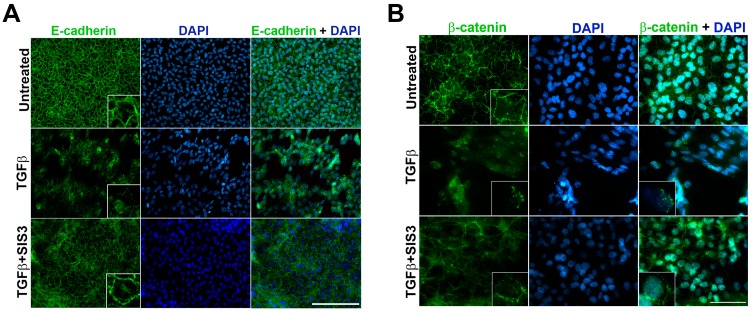
Localization of β-catenin and E-cadherin upon Smad3 inhibition. Rat lens explants were treated with TGF-β in the presence or absence of SIS3 for 48 h (*n* = 3 where *n* ≥ 5 explants per treatment). PFA fixed explants were stained for (**A**) β-catenin and (**B**) E-cadherin and mounted with DAPI to visualize nuclei. Images were acquired using 40X lens of Leica DM6 fluorescence microscope. Scale bars set to 100 µm. Inset shows partial inhibition of β-catenin delocalization (panel 3, B) but complete inhibition of E-cadherin delocalization (panel 3, A) in lens explants co-treated with TGF-β and SIS3.

The E-cadherin/β-catenin complex provides a direct link between cell–cell adhesion complexes and the actin cytoskeleton dislodging of this complex is believed to be a signature of EMT. In the lens, we have previously shown that both E-cadherin and β-catenin primarily localize to the cell peripheries, and stimulation with TGF-β results in delocalization of this complex leading to nuclear translocation of β-catenin [32]. Therefore, we were interested in investigating the effects of inhibition of Smad3 activation on TGF-β-induced delocalization of E-cadherin/β-catenin. As expected, it was found that TGF-β treatment resulted in the loss of E-cadherin from the cell border, exhibiting a disorganized pattern (Figure 2A, panel 2). In comparison, untreated LECs showed membranous localization of E-cadherin outlining the hexagonal shape of the epithelial cells (Figure 2A, inset—panel 1). Interestingly, co-incubation of the LECs with SIS3 and TGF-β prevented E-cadherin loss from the membrane and showed staining similar to that of the untreated LECs (Figure 2A, inset—panel 3). Next, we assessed the localization of β-catenin, the other important signaling molecule in the E-cadherin/β-catenin complex, upon inhibition of Smad3 activation. Immunofluorescence analyses reveal membranous localization of β-catenin in untreated LECs (Figure 2B, inset—panel 1), similar to E-cadherin staining. In contrast, TGF-β treatment showed loss of β-catenin from the cell membrane and an increased nuclear translocation of β-catenin (Figure 2B, inset—panel 2). In comparison to TGF-β-treated LECs, TGF-β and SIS3 co-treated LECs showed a partial rescue of TGF-β-induced delocalization of β-catenin from cell periphery along with a concomitant decrease in nuclear β-catenin indicating an important role for Smad3 in β-catenin delocalization during TGF-β-induced EMT in the lens (Figure 2B, inset—panel 3). 

### 2.3. β-Catenin Inhibition Blocks Smad3 Activation and Nuclear Localization 

**Figure 3:**
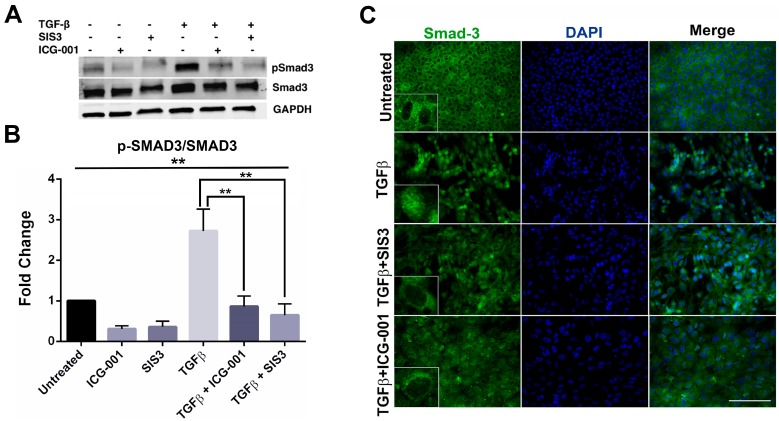
Inhibition of β-catenin signaling blocks activation and delocalization of Smad3. (**A**) Western blot analyses was performed using protein isolated from rat lens explants incubated with TGF-β in the presence or absence of SIS3 or ICG-001 (*n* = 3 where *n* ≥ 6 explants per treatment) and probed for Smad3 and pSmad3, with GAPDH used as a loading control. (**B**) Graph showing the statistical analysis of western blot band densities for pSmad3 compared to Smad3 normalized with the loading control (GAPDH) with ± standard deviation (GAPDH) (*n* = 3, *p* < 0.01) (**C**) Rat lens explants were treated with TGF-β alone (panel 2) and in the presence of SIS3 (panel 3) or ICG-001 (panel 4) for 48 h (*n* = 3 where *n* ≥ 5 explants per treatment). The explants were then fixed and stained for Smad3 and mounted with DAPI to visualize nuclei. The images were acquired using 40× lens of Leica DM6 fluorescence microscope. Insets show inhibition of TGF-β-induced nuclear Smad3 in the presence of either SIS3 or ICG-001. Scale bars set to 100 µm.

The primary response from the interaction of TGF-β with its receptors is phosphorylation-mediated activation, and nuclear translocation, of Smad3 in a complex with Smad2 and Smad4 leading to activation of EMT-related genes [26]. Specifically, phosphorylation of Smad3 at Ser-423/425 has been reported to be critical in translocation of Smad3 to the nucleus, and thus in EMT [26,38]. In order to ascertain the role of β-catenin in TGF-β-induced phosphorylation, and thus activation, of Smad3, we performed western blot analyses for Smad3 and its active form, phosphorylated Smad3 (p-423/425-Smad3) using protein isolated from LECs treated with TGF-β in the presence or absence of SIS3 or ICG-001 (*n* = 3 where *n* ≥ 6 explants per sample). Little or no change in the Smad3 expression was observed among the samples when compared to the respective loading control (Figure 3A). However, a 3-fold increase in the levels of p-Smad3 was observed in the LECs incubated with TGF-β when compared to untreated LECs (Figure 3A). As expected, co-treatment of lens explant with TGF-β and SIS3 resulted in inhibition of TGF-β-induced Smad3 activation (~5-fold decrease). Interestingly, LECs co-incubated with TGF-β and ICG-001 also showed a noticeable decrease (~4-fold) in Smad-induced activation when compared to the LECs treated with TGF-β alone (Figure 3A). 

To determine the fraction of total Smad3 that is being activated, we calculated the ratio of p-Smad3 and Smad3 (p-Smad3 over Smad3) after normalizing the Smad3 western blot bands with GAPDH, the loading control. The graph in Figure 3B shows an ~3-fold increase in the ratio of p-Smad3/Smad3 in the LECs incubated with TGF-β compared to the untreated LECs (Figure 3B, *p* < 0.01; *n* = 3 where *n* ≥ 6 explants per treatment) indicating an overall increase in active Smad3 in the presence of TGF-β. Surprisingly, inhibition of β-catenin activation by ICG-001 prevented TGF-β-induced Smad3 activation. Our analyses reveal a ~3-fold and a ~6-fold decrease in the p-Smad3/Smad3 ratio in the LECs co-treated with TGF-β and ICG-001 and TGF-β and SIS3, respectively, compared to the LECs treated with TGF-β alone (Figure 3B, *p* < 0.01; *n* = 3 where *n* ≥ 6 explants per treatment). This shows an overall decrease in Smad3 activation upon inhibition of β-catenin signaling and Smad3 activation.

As activation of Smad3 is coupled to its nuclear translocation, we next set out to investigate the localization of Smad3. Treatment of LECs with TGF-β resulted in nuclear translocation of Smad3 (Figure 3C, inset—panel 2) compared to the cytoplasmic localization of Smad3 in untreated LECs (Figure 3C, inset—panel 1; *n*=3 where *n* ≥ 5 explants per treatment). As expected, co-incubation of explants with TGF-β and SIS3, the Smad3 activation inhibitor, prevented Smad3 nuclear translocation (Figure 3C, inset—panel 3; *n* = 3 where *n* ≥ 5 explants per treatment). Interestingly, treatment of LECs with ICG-001 prevented TGF-β-induced Smad3 nuclear translocation (Figure 3C, inset—panel 4; *n* = 3 where *n* ≥ 5 explants per treatment), similar to untreated explants, indicating that β-catenin is necessary for Smad3 activation and nuclear localization.

### 2.4. β-Catenin and Smad3 Inhibition Decreases TGF-B Receptor II mRNA Levels

**Figure 4:**
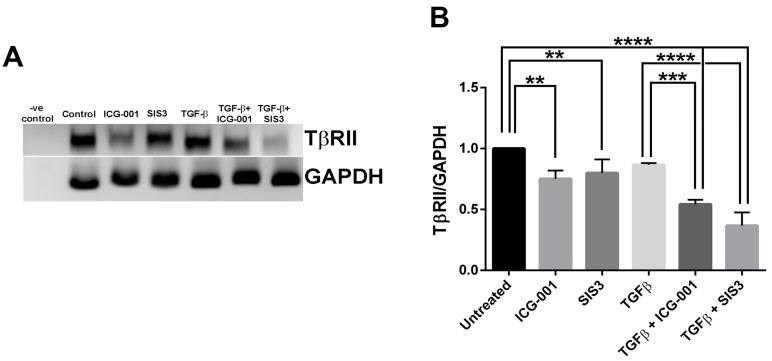
Reverse transcriptase PCR (RT–PCR) for TβRII, and GAPDH. cDNA transcribed from RNA isolated from lens explants incubated with TGF-β in the presence or absence of SIS3 or ICG-001 were amplified using PCR, then visualized on a 1.5% agarose gel by electrophoresis, using GAPDH as a standard control. (**A**) Representative image of the RT–PCR gel showing mRNA levels of TβRII and GAPDH. The PCR sample without cDNA served as a negative control. (**B**) Graph showing statistical analysis (ANOVA–Tukey’s multiple comparisons test) of band densities for TβRII normalized to respective GAPDH. Error bars indicate standard deviation. *p* < 0.01 for untreated vs. ICG-001- and SIS3 –treated LECS; *p* < 0.0001 for untreated vs. TGF-β + SIS3 and TGF-β + ICG-001 co-treated LECs; *p* < 0.0001 for TGF-β-treated vs. TGF-β + SIS3 co-treated LECs; *p* < 0.001 for TGF-β vs. TGF-β + ICG-001 co-treated LECs.

The results outlined in the previous section revealed inhibition of Smad3 activation, but not Smad3 expression levels upon inhibition of β-catenin signaling in the LECs (Figure 3). One possibility for the inhibition of Smad3 activation upon inhibition of β-catenin signaling could be due to the modulation of TGF-β receptors’ expression by β-catenin signaling [39]. In order to test this hypothesis, we performed semi-quantitative reverse-transcriptase (RT)–PCR for TGF-β receptors I (TβRI) and II (TβRII). Relative expression levels of TβRI, TβRII and GAPDH (experimental control) were calculated using ImageJ software and statistical analyses (ANOVA–Tukey’s multiple comparisons test) on the relative expression levels were carried out using GraphPad Prism 6 software. The statistical analyses did not show any significant change in the levels of TβRI among various treatment groups (unpublished observation. However, the mRNA levels of TβRII showed significant differences between various treatment groups. Figure 4A represents one of the gels (*n* = 3) considered for statistical analyses for TβRII and GAPDH (Figure S1 for full gel image). The LECs treated with TGF-β in the presence of ICG-001 show a significant reduction in mRNA levels for TβRII compared to untreated (**** *p* < 0.0001) or TGF-β-treated (*** *p* < 0.001) LECs (Figure 4B). Furthermore, co-incubation of LECs with TGF-β and SIS3 also showed a significant decrease in the levels of mRNA transcript for TβRII compared to either untreated or TGF-β-treated LECs (Figure 4B; **** *p* < 0.0001). However, we did not observe any significant difference between LECs treated with TGF-β + ICG-001 and TGF-β + SIS3.

### 2.5. Smad3 Activation Inhibition Blocks MRTF-A Delocalization

**Figure 5:**
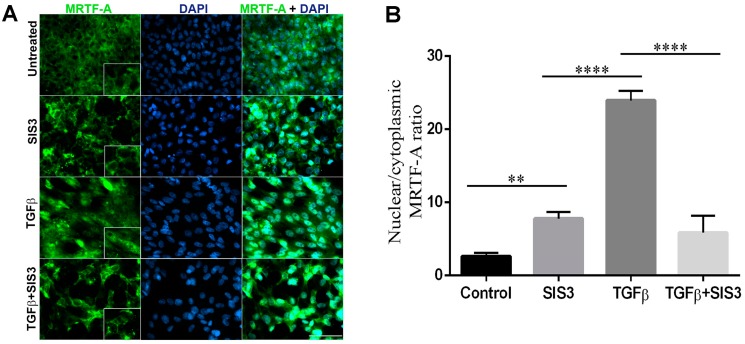
Inhibition of Smad3 activation prevents nuclear translocation of MRTF-A. (**A**) Rat lens explants were treated with TGF-β in the presence or absence of SIS3 for 48 h (*n* = 3 where *n* ≥ 5 explants per treatment). Fixed explants were stained for MRTF-A and mounted with DAPI to visualize nuclei. The images were acquired using the 40× lens of the Leica DM6 fluorescence microscope. Inset clearly shows inhibition of TGF-β-induced nuclear MRTF-A in the presence of SIS3. Scale bars set to 100 µm. (**B**) A bar graph depicting the quantified ratio of the nuclear-to-cytoplasmic MRTF-A signal by measuring the intensity of fluorescence in the images as described in Materials and Methods. Statistical analysis was performed using GraphPad Prism 6. Data expressed as the means ± SD from the three separate experiments.

The interaction of Smad3 with MRTF-A, an actin-binding protein and a downstream regulator of Rho/ROCK signaling, has been shown to be critical during EMT-induced fibrosis of the lungs and kidneys [33,35]. In the lens explant system, we have previously shown that MRTF-A translocates to the nucleus upon TGF-β stimulation [40] and inhibition of MRTF-A nuclear translocation prevents TGF-β-induced EMT [31]. In order to understand the role of Smad3 in TGF-β-induced MRTF-A nuclear translocation, we performed immunofluorescence analysis on LECs incubated with TGF-β in the presence or absence of SIS3. In untreated or SIS3 treated LECs MRTF-A was observed to be non-nuclear and localized to the cytoplasm (Figure 5A, panel 1 and 2, *n* = 3 where *n* ≥ 5 per treatment). As shown previously [40], incubation of LECs with TGF-β resulted in nuclear translocation of MRTF-A (Figure 5A, panel 3, *n* = 3 where *n* ≥ 5 per treatment). The TGF-β-induced translocation of MRTF-A was inhibited in the presence of SIS3 (Figure 5A, panel 4, *n* = 3 where *n* ≥ 5 per treatment) and was observed to be localized in the cytoplasm. In order to demonstrate the nuclear vs. cytoplasmic localization of MRTF-A, we quantified the ratio of nuclear-to-cytoplasmic fluorescence intensity of MRTF-A across the lens explant treatment groups using ImageJ software. Our quantification analyses show a 21.3-fold higher nuclear-to-cytoplasmic MRTF-A ratio in TGF-β-treated LECs when compared to untreated controls (*p* < 0.0001; Figure 5B). In contrast, MRTF-A nuclear-to-cytoplasmic fluorescence intensity with TGF-β treatment in the presence of SIS3 was 18.1-fold lower than explants treated with TGF-β alone (*p* < 0.0001; Figure 5B). These observations demonstrate the role of Smad3 in regulating the localization of MRTF-A, and thus lens EMT.

## 3. Discussion

Understanding the mechanism of TGF-β-induced molecular signaling events is key to developing novel therapeutic strategies for the prevention of EMT-induced fibrosis of the ocular lens, thus preventing cataract. Our laboratory, and others, have demonstrated that molecules such as β-catenin, Smad3, and MRTF-A are critical regulators of TGF-β-induced EMT in the lens [31,32,41]. However, the interaction between these molecules has not been fully addressed. In the present study, we investigated the importance of cross-talk between these key signaling molecules; β-catenin, Smad3 and MRTF-A, during TGF-β induced EMT using the rat lens explant system. In particular, we have shown that inhibition of β-catenin signaling by ICG-001, an inhibitor of β-catenin/CBP interaction, resulted in inhibition of TGF-β-induced Smad3 activation. Similarly, inhibition of Smad3 activation by SIS3, an inhibitor of Smad3 activation, was able to partially prevent TGF-β-induced β-catenin delocalization from the lens epithelial cell membrane. 

Canonically, TGF-β activates Smad2 and Smad3 through direct phosphorylation of its type I TGF-β receptor. The phosphorylated Smad2/Smad3 complex then forms a trimeric complex with Smad4, and translocates to the nucleus, where it associates with DNA binding transcription factors to modulate the expression of target genes such as α-SMA [26,42]. In the current study we showed that co-treatment with SIS3, an inhibitor of Smad3 activation, resulted in inhibition of TGF-β-induced α-SMA expression and stress fiber formation in LECs. This is in agreement with our previous report in mice in which knocking out Smad3 expression on a TGF-β transgenic background led to decreased expression of TGF-β-mediated EMT markers such as α-SMA, fibronectin and collagen I and IV [30]. Disruption of E-cadherin/ β-catenin, and subsequent degradation of E-cadherin is another hallmark of TGF-β-induced EMT in the lens. The prevention of TGF-β-induced E-cadherin membrane delocalization, and preservation of the hexagonal shape of lens epithelial cells resembling that of untreated cells, indicates the role of TGF-β/Smad3 signaling in regulation of E-cadherin in the lens. In addition, partial prevention of TGF-β-induced membrane delocalization of β-catenin, and its subsequent nuclear translocation, in the presence of SIS3 (Figure 2) reveals the role of Smad3 signaling in indirectly regulating β-catenin signaling during TGF-β-induced EMT in LECs. These observations are in agreement with a previous report where interaction of TGF-β with TGF-β receptor II/Smad complex resulted in stress-fiber-mediated disassembly of adherens junctions leading to increased membrane delocalization of E-cadherin and stabilization of the cytoplasmic pool of β-catenin [28]. However, it is possible that TGF-β-induced loss of β-catenin localization is also regulated by Smad-independent mechanism/s since the restoration of β-catenin to the lens epithelial cell membrane in the presence of TGF-β and SIS3 was partial. 

TGF-β-induced Smad signaling has also been shown to cross-talk with other pathways, such as the Wnt/β-catenin [39]. For example, during EMT in other systems such as the kidney and lungs, β-catenin has been shown to form a complex with Smad3, which then translocates to the nucleus, cooperatively promoting the transcription of EMT target genes [33,34,35]. Interestingly, in the current study, we found that in LECs co-treated with TGF-β and the inhibitor of β-catenin/CBP signaling, ICG-001, Smad3 activation and nuclear translocation was suppressed indicating a novel role of β-catenin signaling in Smad3 activation during TGF-β-induced EMT in LECs (Figure 3). One possible mechanism for the decrease in TGF-β-induced Smad3 activation upon β-catenin/CBP signaling inhibition could be due to increased activity of inhibitory Smads such as Smad-7. For example, a previous report suggested that Smad-7 directly binds to β-catenin and promotes β-catenin degradation thereby reducing β-catenin signaling [43]. However, a recent study from our laboratory has shown that while inhibition of β-catenin/CBP signaling by ICG-001 prevents TGF-β-induced membrane delocalization of β-catenin, it did not lead to its degradation [32], thus suggesting that ICG-001 does not control Smad3 in this way. Another possibility is that ICG-001 regulates the expression of the TGF-β receptors that are required for Smad3 activation. Indeed we did find, using reverse transcription (RT)–PCR assay, that TβRII mRNA levels were decreased in LECs co-treated with ICG-001 and TGF-β, as compared to those treated with TGF-β alone (Fig. 4). A detailed in-silico analysis for TGF-β receptor I and II promoter reveal *cis* binding sites for CREB1 [44], a transcription factor that requires the transcriptional co-activator CBP, a known interacting partner of β-catenin during EMT [45,46], for its transcriptional activity [47,48]. Therefore, it is possible that inhibition of β-catenin/CBP interaction by ICG-001 in LECs might be negatively regulating the transcriptional activity of CREB1, and thus TβRII expression. Indeed, further detailed molecular investigations are required to demonstrate the nature of the interaction between β-catenin/CBP and CREB1 in regulation of TGF-β receptor expression during TGF-β-induced EMT in LECs. 

Previous studies on TGF-β-induced EMT in the lens have mostly focused on canonical pathways of signaling involving Smads [42]; however, non-canonical pathways such as β-catenin/RhoA/MRTF-A, and its cross-talk with canonical TGF-β/Smad pathways, have also been shown to be critical during TGF-β induced EMT [31,32,33,34,49]. In the context of lens EMT, we have shown that TGF-β induces Rho-A-mediated MRTF-A nuclear translocation, in addition to β-catenin nuclear translocation, which leads to induction of α-SMA expression [31]. Furthermore, we have shown that direct inhibition of MRTF-A nuclear translocation in LECs prevents TGFβ-induced EMT in LECs [31]. The inhibition of TGF-β-induced MRTF-A nuclear translocation, and subsequent reduction in TGF-β-induced α-SMA expression and stress fiber formation, in the presence of SIS3 highlights the intimate connection between canonical and non-canonical signaling during TGF-β-regulated EMT in the lens. This inference is in conjunction with a previous report where interaction of Smad3 with MRTF-A has been shown to regulate MRTF-A nuclear translocation, and thus α-SMA expression [33]. Indeed, further investigations are needed to elucidate the nature of interactions between Smad3 and MRTF-A, which regulates MRTF-A nuclear translocation and α-SMA expression in the lens. 

**Figure 6:**
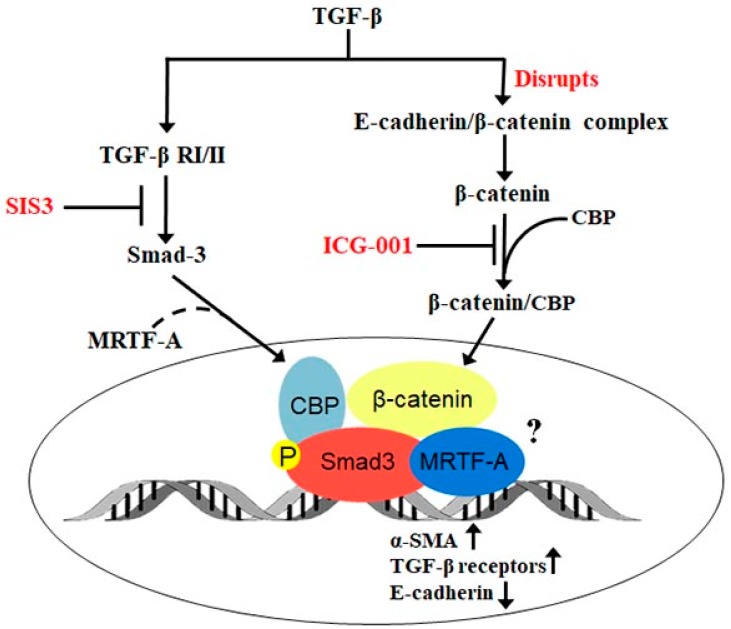
Schematic representation of the β-catenin and Smad3 interaction-mediated regulation of TGF-β-induced EMT in LECs. TGF-β results in activation of both canonical (Smad3) and non-canonical (β-catenin) signaling. Upon TGF-β-induced disruption of E-cadherin/β-catenin, free β-catenin interacts with CBP and translocates to the nucleus. TGF-β-induced active Smad3 translocates to the nucleus and also regulates nuclear translocation of MRTF-A possibly through formation of the Smad3/MRTF-A complex. β-catenin/CBP might form a complex with Smad3 and MRTF-A in the nucleus, which might then regulate EMT-related genes.

Our present findings reveal that an integrated signaling network, as opposed to an individual signaling pathway is required to regulate TGF-β-induced EMT in the lens. We propose that both β-catenin- and Smad- mediated signaling is important during TGF-β-induced EMT in the lens, and that blocking either pathway negatively affects the other. We show that active Smad3 regulates disruption of the E-cadherin/β-catenin complex, and thus β-catenin nuclear localization and lens EMT. This may occur through modulation of TGF-β-induced E-cadherin expression [50], or be due to disassembly of adherens junctions by the TGF-β/Smad complex [28], or both. We demonstrate for the first time that β-catenin/CBP-dependent signaling regulates activation of Smad3, possibly through modulation of TGF-β receptors, in the lens. The interaction between β-catenin and Smad3 is known to promote transcriptional activity of MRTF-A, the master regulator of cytoskeletal genes, during TGF-β-induced EMT in tubular epithelial cells [33]. Interestingly, our previous findings suggest that nuclear MRTF-A is critical for TGF-β-induced EMT in the lens [31,40]. The inability of MRTF-A to translocate to the nucleus in the presence of SIS3 and modulate α-SMA expression reveals that Smad3 is an upstream regulator of nuclear translocation of MRTF-A in the lens (Figure 6). In addition, prevention of Smad3-mediated nuclear MRTF-A by ICG-001 in the presence of TGF-β shows that β-catenin is a key regulator of both β-catenin/CBP and Smad-3/MRTF-A signaling during TGF-β-induced EMT in the lens. Taken together, using our previous and current findings, we propose that the interplay between β-catenin, Smad3 and MRTF-A is crucial for TGF-β-mediated EMT in the lens. Thus, the inhibition of multiple pathways using a combinatorial approach rather than blocking a single pathway during TGF-β induced EMT in the lens might prove to be efficient against ocular fibrosis of the lens.

## 4. Materials and Methods 

### 4.1. Reagents

Recombinant human TGF-β2 was obtained from R&D Systems (Minneapolis, MN, USA). Inhibitors SIS3 and ICG-001 were purchased from Sigma-Aldrich (St. Louis, MO, USA) and Calbiochem-EMD millipore (Temecula, CA, USA), respectively. Primary antibodies included E-cadherin from BD Transduction Laboratories (Lexington, KY, USA), active β-catenin (clone 8E7) from Upstate (Lake Placid, NY, USA), GAPDH from Abcam (Cambridge, MA, USA), αSMA fluorescein isothiocyanate (FITC)-conjugated and unconjugated from Sigma-Aldrich. All secondary antibodies for immunofluorescence staining and Rhodamine Phalloidin were purchased from Molecular Probes (Invitrogen, Carlsbad, CA, USA); secondary antibodies for Western blots were obtained from LI-COR Biosciences (Lincoln, NE, USA). 

### 4.2. Culturing Rat Lens Epithelial Explants

The major technique used in the lab is the explanting of lens epithelium (LECs) on its native basement membrane to create ex vivo models of LECs for observation of EMT. The eyes of juvenile rats between the ages of 19–21 days were removed after euthanasia via CO_2_ overdose followed by cervical dislocation. Eyes were placed in M199 media (Gibco by Life Technologies, Gaithersburg, MD, USA) and warmed to around 37 °C. Under a dissecting microscope (Leica MZ16, manufacturer, Concord, ON, Canada) the capsular bag of the eye was ripped open using two teethed forceps and all structures excluding the lens were discarded and lenses were then transferred to fresh plates of media. Two sharp-pointed forceps were used to peel off the thin layer of epithelial cells on the posterior side of the lens by creating tears in the anterior side indicated by striations in the tissue. The epithelial cells on the anterior layer were then carefully peeled off and pinned to the plate with a blunt tool. The LECs were stored in an incubator at 37 °C at 5% CO_2_, and 95% humidity until the next day.

### 4.3. Treatment of Lecs with TGF-B and Inhibitors

Explants were either left untreated or treated with SIS3 (10uM) or ICG (10uM) (Sigma-Aldrich Corp, St Louis, MO, USA) in 2mL of media and were incubated for 1 hour. Indicated plates were then co-treated with 6ng/mL of TGF-β and incubated for 48 h. Unless otherwise mentioned all treatments were done for 48 h.

### 4.4. Western Blot Analyses

LECs (*n* > 5–7 explants per treatment) were collected and lysed in a mixture of protease phosphatase inhibitor (Roche Diagnostics, Germany) and lysis buffer (50 mM Tris-HCl pH 8.0, 150 mM NaCl, 1% Triton-X-100) and stored at −20 °C. Protein concentrations from each sample were estimated using the DC Protein Assay in a microplate (Bio-rad, Hercules, CA, USA). Next, 10 µg of protein samples were processed and prepared for loading and loaded into the polyacrylamide gel and SDS–PAGE was performed. Resolved bands were transferred onto a polyvinylidene difluoride PVDF membrane (Bio-rad). Ponceau was used to stain the blot in order to check for protein presence. Ponceau stained blots were washed with TBS and then blocked with Odyssey Blocking Buffer (PBS) (LI-COR Biosciences, Lincoln, NE, USA) for 50 minutes to an hour with gentle rocking. Specific proteins were probed for by incubating the membrane with primary antibody, either GAPDH (Abcam, Cambridge, MA, USA) or α-SMA (Sigma-Aldrich) at a dilution of 1:10,000, or p-Smad Ser-423/425 (Cell Signaling, Danvers, MA, USA) or Smad3 (Abcam) at a dilution of 1:500, and diluted with Tris-buffered saline and Tween-20 (TBST) overnight at 4 °C. After, the membrane was washed 3 times for 10 min each in TBST with gentle rocking. Corresponding secondary antibodies were diluted with TBST to make a 1:10,000 dilution and added to the membrane to incubate for 1 h. The membrane was then washed with TBST 3 times for 10 min each, scanned, and visualized using the Odyssey near-infrared scanning system (LI-COR Biosciences). The band densities were analyzed using ImageJ software and the readings were normalized with their respective control bands (GAPDH). The final readings acquired from three independent experiments (*n* = 3) were analyzed using ANOVA and graphed using GraphPad Prism 6.

### 4.5. Immunocytochemistry

LECs with various treatments were fixed with 10% neutral buffered formalin for a total of 15 min, washed with phosphate-buffered saline solution (PBS; Invitrogen), then lifted from the culture dish surface using a blunt single-pronged tool and placed into separate test tubes with PBS. The LECs were blocked and permeabilized with 5% normal donkey serum (NDS; Invitrogen) in a permeabilizing buffer (0.1% Triton-X-100 and 0.5% sodium dodecyl sulphate (SDS) in PSB) for 1 h at room temperature with gentle rocking. Next, the LECs were incubated with primary antibody overnight, which was either Smad3 (Abcam), MRTF-A (Santa Cruz Biotechnology, Dallas, TE, USA), α-SMA (Sigma-Aldrich), or E-cadherin (Santa Cruz Biotechnology), all at dilutions of 1:200 in PBS at 4 °C. The LECs were washed 3 times for 10 min each with PBS solution before adding the corresponding secondary antibody (Life Technologies), at a concentration of 1:200, after which they were covered and incubated at room temperature for 1 hour with gentle rocking. Stained LECs were then mounted onto slides with 3 LECs per glass slide and mounted with Prolong Gold antifade reagent with 4’-6-diamidino-2-phenylindole (DAPI, Invitrogen, Life Technologies) in order to visualize the nucleus. Fluorescent staining was visualized with a Leica fluorescent microscope (DM6 B, Leica, Germany) and images captured using a Leica CTR6 LED camera. MRTF-A nuclear localization was analyzed using Fiji image processing software [51], where nuclear MRTF-A fluorescence intensity was measured and normalized to cytoplasmic fluorescence. Nuclear/cytoplasmic ratios were reported relative to untreated control ratios. 

### 4.6. Reverse Transcription (RT)–PCR Assay

LECs (*n* ≥ 5 per sample) with various treatments were collected and stored in lysis buffer at –20 °C. RNA was isolated according to the RNeasy Mini Kit (Qiagen, Hilden, Germany) with sonication proceeding isolation. The concentration of RNA for each sample was determined with the Nanodrop 2000 (ThermoFisher Scientific, Carlsbad, CA, USA). Next, 430–500 ng/µL of RNA per sample was used to create cDNA following the protocol from the High-Capacity RNA-to-cDNA Kit (ThermoFisher Scientific). PCR was carried out using 1uL of forward and 1uL of reverse primer of four separate primers including TβRII and GAPDH (Table S1 for primer sequences; TβRI primer sequences included). PCR products were electrophoresed on a 1.5% agarose gel for 1 h then visualized using GenSys. The PCR sample without cDNA served as a negative control. The TβRII bands normalized to its respective GAPDH bands were then analyzed using ImageJ software and statistical analysis (one-way ANOVA–Tukey’s multiple comparisons test) was done using GraphPad Prism 6 software. The error bars denote standard deviation (SD).

## Supplementary Materials

Supplementary materials can be found at https://www.mdpi.com/1422-0067/20/9/2078/s1.
