# Supplementary material for: β-Catenin/Smad3 Interaction Regulates Transforming Growth Factor-β-Induced Epithelial to Mesenchymal Transition in the Lens
Source: Int J Mol Sci. 2019 Apr 27;20(9):2078. doi: 10.3390/ijms20092078 (PMC6540099; doi:10.3390/ijms20092078)
Supplement: Supplementary file 1 [file ijms-20-02078-s001.pdf]

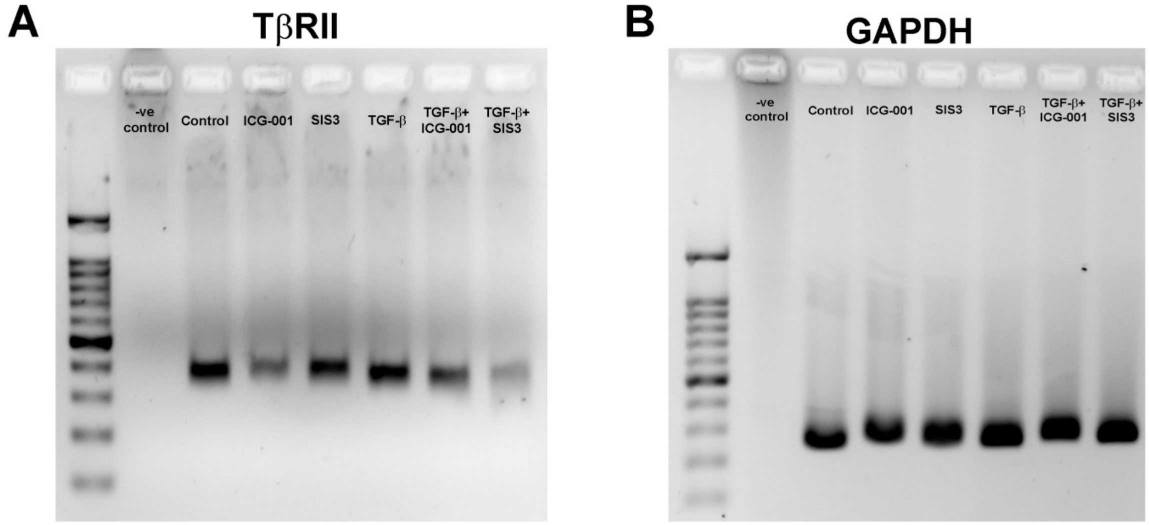

Figure S1. Gel images for Reverse Transcriptase (RT-PCR) for TβRII and GAPDH. cDNA transcribed from RNA isolated from lens explants incubated with TGF-β in the presence or absence of SIS3 or ICG-001 were amplified using PCR, then visualized on a 1.5% agarose gel by electrophoresis, using GAPDH as a standard control. The PCR sample without cDNA served as a negative control.
